# Small molecule innate immune modulators in cancer therapy

**DOI:** 10.3389/fimmu.2024.1395655

**Published:** 2024-09-10

**Authors:** Avijit Goswami, Sandeep Goyal, Princy Khurana, Kawaljit Singh, Barnali Deb, Aditya Kulkarni

**Affiliations:** ^1^ Aten Porus Lifesciences Pvt. Ltd., Bengaluru, India; ^2^ Avammune Therapeutics, Philadelphia, PA, United States

**Keywords:** small molecule immunomodulators, cancer immunotherapy, innate immunity, immuno-oncology, small molecule

## Abstract

Immunotherapy has proved to be a breakthrough in cancer treatment. So far, a bulk of the approved/late-stage cancer immunotherapy are antibody-based. Although these antibody-based drugs have demonstrated great promise, a majority of them are limited due to their access to extracellular targets, lack of oral bioavailability, tumor microenvironment penetration, induction of antibody dependent cytotoxicity etc. In recent times, there has been an increased research focus on the development of small molecule immunomodulators since they have the potential to overcome the aforementioned limitations posed by antibodies. Furthermore, while most biologics based therapeutics that are in clinical use are limited to modulating the adaptive immune system, very few clinically approved therapeutic modalities exist that modulate the innate immune system. The innate immune system, which is the body’s first line of defense, has the ability to turn cold tumors hot and synergize strongly with existing adaptive immune modulators. In preclinical studies, small molecule innate immune modulators have demonstrated synergistic efficacy as combination modalities with current standard-of-care immune checkpoint antibodies. In this review, we highlight the recent advances made by small molecule innate immunomodulators in cancer immunotherapy.

## Introduction

Immunotherapy is a type of therapy which uses a patient’s own immune system to fight the disease (1, 2). Recent advances in cancer therapy have highlighted the emergence and frequent use of immunotherapy as an alternative for hard to treat, refractory/relapse cases (3). Current immuno-oncology therapeutics are based on antibodies that target various proteins and receptors. These act as immune checkpoints to block immune activation in the tumor microenvironment (TME) (3–5).

Several monoclonal antibodies that target immune checkpoints are clinically approved for cancer therapy (6). Antibodies targeting immune checkpoints such as programmed cell death-1 (PD-1) and its ligand PD-L1 are now approved for various advanced cancers (7–10). In general, the immunoglobulin backbone poses PK challenges such as long half-life (9-21 days), natural killer mediated antibody-dependent cellular cytotoxicity which increases the chances of adverse events and poor bioactivity (11–14). Lack of oral bioavailability and limited solubility leads to feasibility challenges like maximal administrable volume for the intravenous (IV), intramuscular (IM), subcutaneous (SC) or intratumor (IT) delivery (13, 15). Apart from these concerns, antibody therapy is challenged by poor anti-tumor efficacy in some solid tumors due to lack of cell permeability resulting in the inability to recognize intracellular targets (16).

In contrast to therapeutic antibodies, small molecule immunomodulators have superior drug-like characteristics in terms of pharmacokinetic properties such as oral bioavailability, reasonable half-life, and membrane permeability (17–19). Apart from this, patient adherence is trickier with antibody therapy due to the intravenous administration while small molecules are often administered orally as a pill or tablet form allowing for better patient compliance. Interestingly, various preclinical and clinical studies have reported that small molecule immunomodulators when used as combination modalities with antibody therapy demonstrates synergistic anti-tumor efficacy and also used as adjuvants in cancer treatment (20–23). Surprisingly, majority of the focus has so far been towards harnessing the adaptive immune system by inhibiting the immunosuppressive signaling with immune checkpoint inhibitors. Tumor microenvironment (TME) comprises of multiple components such as the adaptive immune cells like (T and B cells) and innate immune cells like macrophages, dendritic cells, myeloid-derived suppressor cells, neutrophils, natural killer cells. Cell types such as macrophages, dendritic cells and natural killer cells that perform the routine surveillance are the first to encounter cancer cells (24, 25). In this review, we summarize the recent advances in small molecules innate immunomodulators being developed for treating cancer.

## Innate immune system in cancer

**Figure 1:**
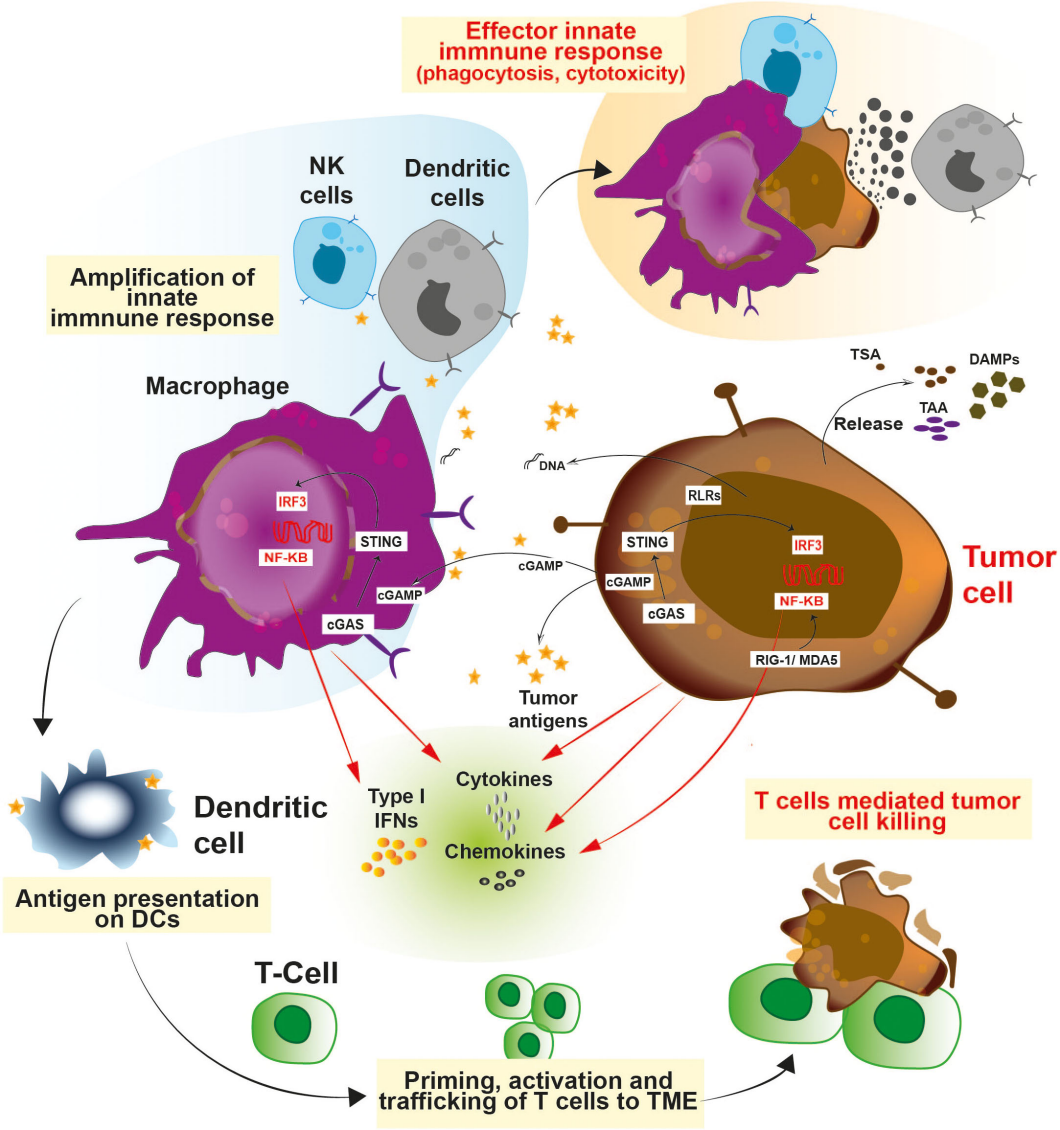
Schematic representation of the various innate immune pathways: Innate immune cells consists of a battery of sensors which equip them to fight against infection and cancer. Activation of STING and RIG-I pathways exerts a robust interferon response which allows the immune system to act against cancer and also curtail infection. Release of type 1 interferons, cytokines and chemokines facilitates the trafficking of the T cells to the TME and primes their activation. The cross-talk between innate and adaptive immune cells enables a potent anti-tumor immunity.

Innate immune cells detects and processes cancer antigens and can directly eradicate tumors by its effector responses such as cytotoxicity and phagocytosis (26–31). Various pathways are involved in triggering the innate immune pathways (**Figure 1**). Antigen presenting cells such as dendritic cells and macrophages utilizes a specific type of DNA sensor known as cGAS/STING pathway. The cGAS/STING pathway senses chromosomal instabilities, double stranded breaks and fragmented DNA in cancer cells (32–34). Tumor derived DNA fragments resulting from genomic instability, mitochondrial dysfunction, and oxidative stress are sensed by the cGAS protein. cGAS converts ATP and GTP into a cyclic dinucleotide (CDN) known as 2’3’-cGAMP, a secondary messenger which acts as a ligand for STING protein and results in activation of the pathway (35–39). Various studies have demonstrated that tumor derived 2′3′-cGAMP molecules act as an immune activator and directly trigger anti-tumor immunity (40–44). Ecto-nucleotide pyrophosphatase/phosphodiesterase-1 (ENPP1/NPP1), a membrane-bound nucleotide hydrolase hydrolyzes 2’3’-cGAMP and dampen the STING-dependent anti-tumor innate immune response (**Figure 1**). ENPP1 acts as a negative regulator for the STING pathway (45). Interestingly, higher expression of ENPP1 has been directly linked with cancer progression, metastasis, and poor response to immunotherapy (46–48).

Besides the cGAS/STING pathway, other immunomodulatory mechanisms involved in cancer include Toll-like receptor (TLRs), RIG-I, NLRP3, and CD47-SIRPα pathways (**Figure 1**) (49–51). TLRs are damage or pattern recognition molecules responsible for immune activation. For e.g., TLR-3, TLR-7 and TLR-9 are widely explored for their role in dendritic cell maturation (52). TLR-7 agonists have demonstrated significant immunostimulatory and anti-tumor efficacy in various cancer models (53). Alternative strategies for igniting the innate immune response and warming up cold tumors include RIG-I and NLRP3 (54, 55). MDA5 and RIG-I have different preferences for RNA ligands, while having comparable structures and causes upregulation of type I interferon signaling (54, 55). Endogenous RNAs are modified to escape the response triggered by the RNA sensors present in the cytosol. Adenine to Inosine conversion is one such modification catalyzed by the protein Adenosine deaminase acting on RNA (ADAR) (56). Tumors with high interferon signature are ADAR1 dependent, and transcripts induced by interferon contributes to accumulation of dsRNA in the cytoplasm (57). ADAR1 depletion resulted in upregulation of interferon stimulated genes (ISGs) in a RIG-1, MDA5 dependent manner (58, 59). Further, deletion of ADAR1 also rendered these cells more prone to immuno-therapy and overcame resistance to immune checkpoint blockade (60), demonstrating ADAR1 as a new immuno-oncology target. Apart from the RIG-I pathway, the NLRP3 inflammasome is a pattern recognition receptor which is activated by both exogenous and endogenous stimuli, leading to a downstream inflammatory response (61, 62). Reports have shown that NLRP3 inflammasomes are necessary for anti-tumor effect during radiation (61). Upon activation, NLRP3 triggers IL-1 mediated maturation of dendritic cells resulting in cross-priming and T-cell immune response (63). Cancer cells escape the innate immune response by means of SIRPα-CD47 interaction which provides a ‘‘don’t-eat-me’’ signal to the effector immune cells like neutrophils and macrophages (64). Increasing evidence suggests that SIRPα-CD47 immune checkpoint blockade enhances the efficacy of cancer immunotherapy (65–68). In this review, we discuss about various small molecule modulators (Summarized in **Table 1**) of targets such as STING/ENPP1, TLR, NLRP3, and SIRPα-CD47 which are key players in the innate immune system working against cancer.

**Table 1:** List of innate immune modulators in cancer immunotherapy.

STING AGONISTS			
ID	Structure	Indication	Reference
**DMXAA** **(Non-CDN analog)**	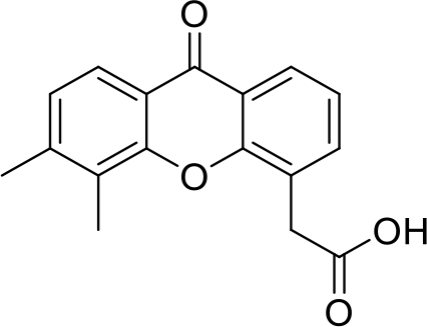	Advanced urothelial carcinoma (Phase II, withdrawn); Non-small cell lung cancer (Phase III, terminated)	NCT01071928 NCT00738387;
**ADU-S100** **(CDN analog)**	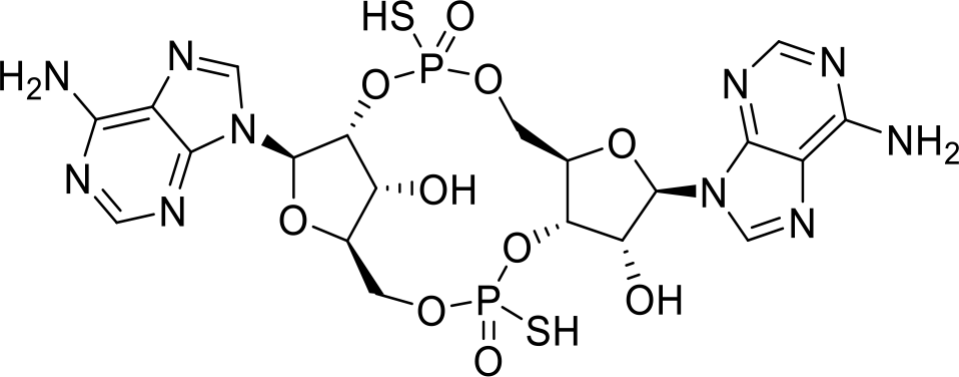	Advanced/metastatic solid tumors or lymphomas (Phase Ib; Terminated) NCT02675439 NCT03172936	(69) (70)
**TAK-676** **(CDN analog)**	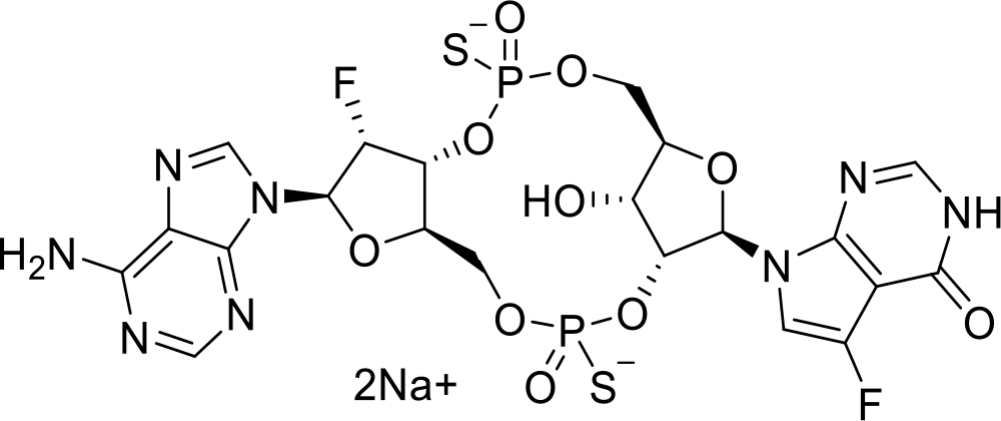	Solid tumors (Phase I) NCT04879849	(71)
**Ulevostinag** **(MK1454)** **(CDN analog)**	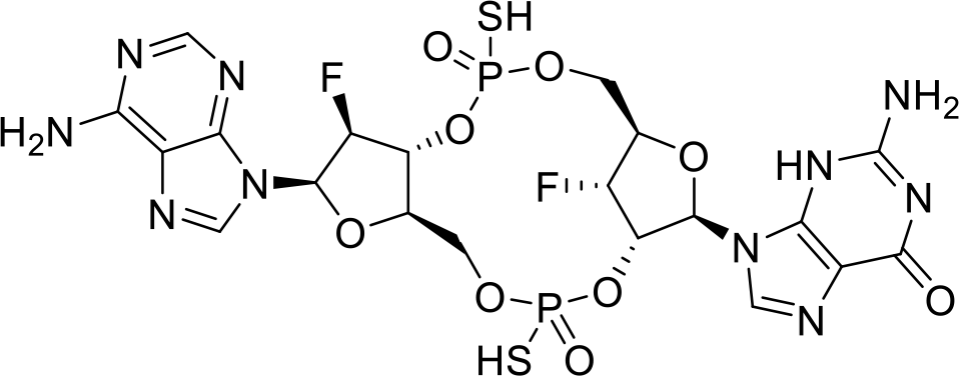	Advanced/metastatic solid tumors or lymphomas (Phase II) NCT03010176 NCT04220866	(72)
**BMS986301** **(CDN analog)**	Not disclosed	Advanced solid cancers (Phase I)	NCT03956680
**SB-11285** **(CDN analog)**	Not disclosed	Melanoma, head and neck squamous cell carcinoma, Solid tumor (Phase IA/IB) NCT04096638	(73)
**BI-1387446** **(CDN analog)**	Not disclosed	Advanced solid tumor (Phase I)	NCT04147234
**IMSA-101** **(CDN analog)**	Not disclosed	Advanced treatment refractory malignancies (Phase I/IIA)	NCT04020185
**GSK3745417** **(Non-CDN analog)**	Not disclosed	Leukemia, Myeloid, Acute (Phase I) Neoplasms (Phase I)	NCT05424380 NCT03843359
**SNX281** **(Non-CDN analog)**	Not disclosed	Advanced solid tumors and Lymphoma (Phase I)	NCT04609579
**E7766** **(Non-CDN analog)**	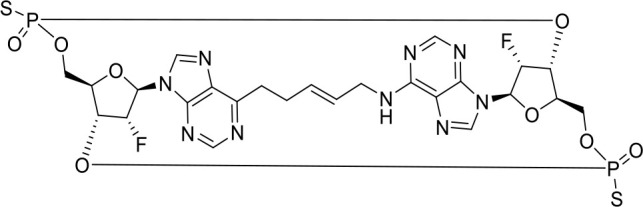	Lymphoma and advanced solid tumors (Phase I/IB) NCT04144140	(74)
**HG-381** **(Non-CDN analog)**	Not disclosed	Advanced solid tumor (Phase I)	NCT04998422
**ABZI** **(Non-CDN analog)**	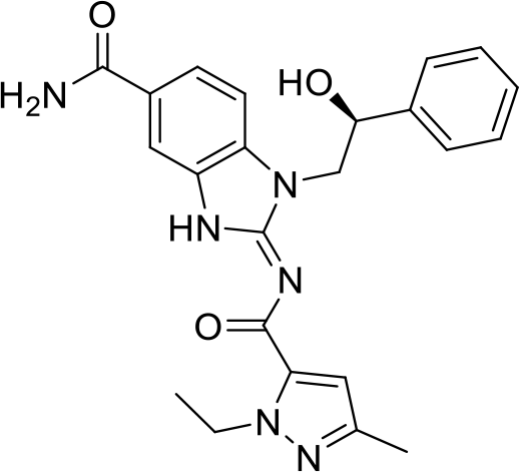	Preclinical	(75)
**di-ABZI** **(Non-CDN analog)**	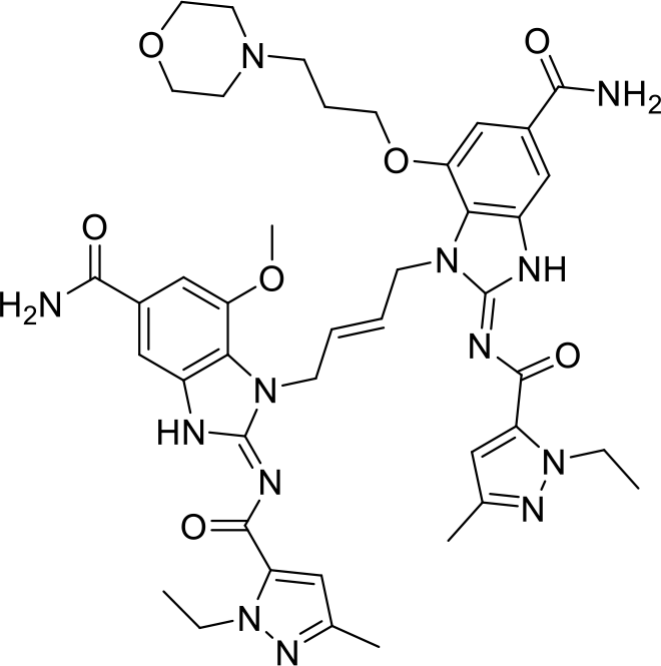	Preclinical	(75)
**MSA-2** **(Non-CDN analog)**	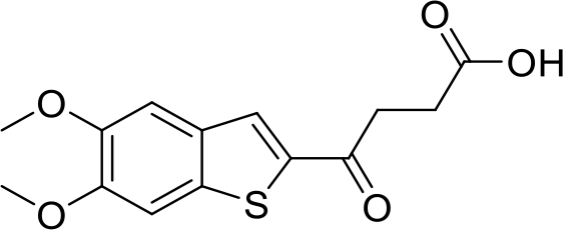	Preclinical	(76)
**MSA-2 Dimer** **(Non-CDN analog)**	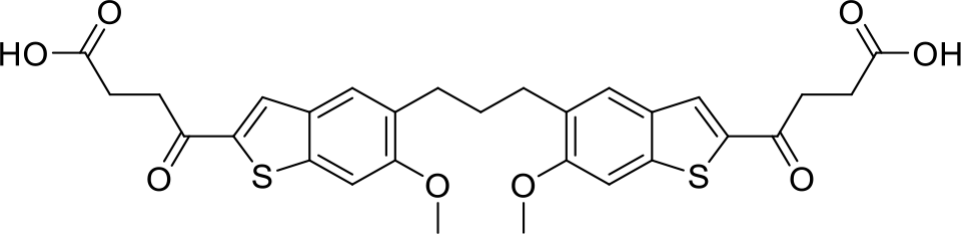	Preclinical	(76)
**SR717** **(Non-CDN analog)**	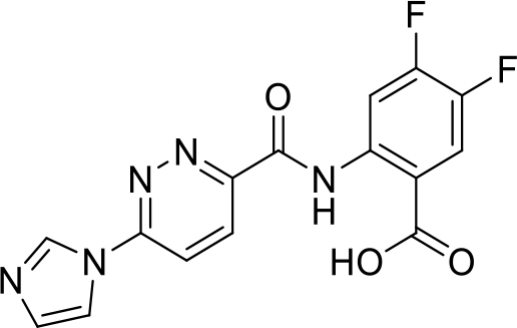	Preclinical	(77)
**CRD5500** **(Non-CDN analog)**	Not disclosed	Preclinical	(78)
**SHR1032** **(Non-CDN analog)**	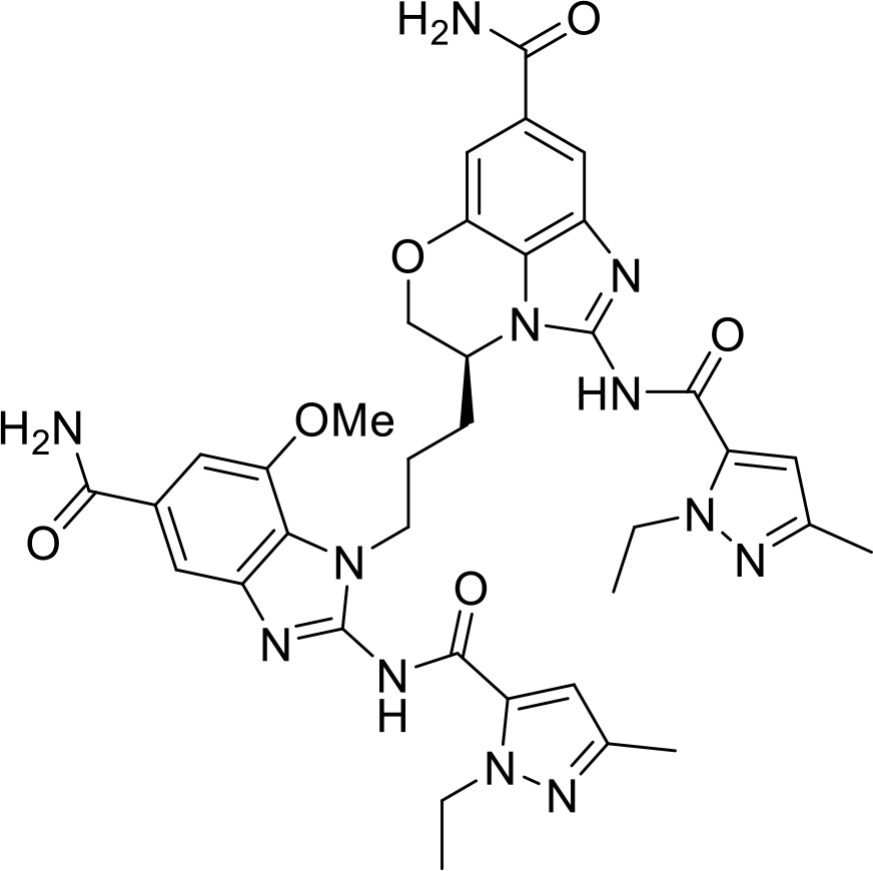	Preclinical	(79)
**αEGFR-172ADC**	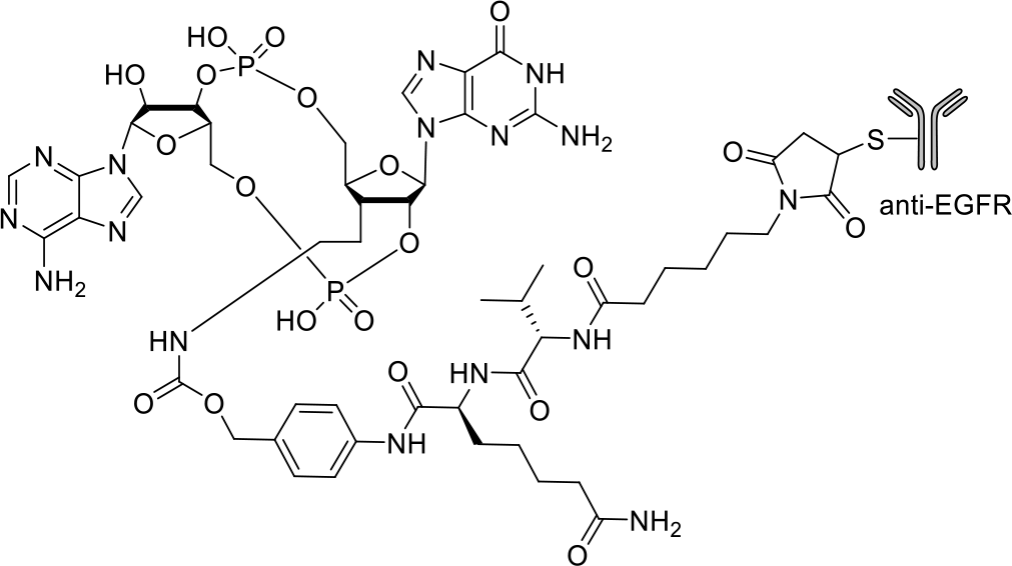	Preclinical	(80)
CD47 inhibitor			
**NCGC00138783**	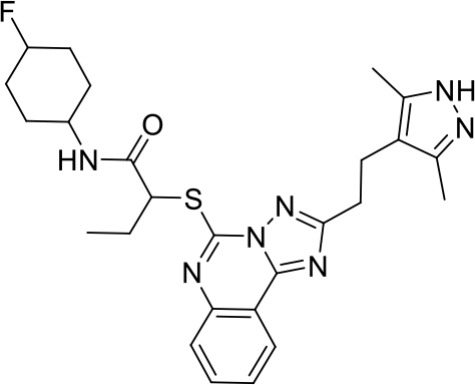	Leukemia (Preclinical)	(81)
**RRx-001**	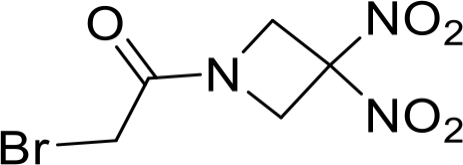	Small cell lung cancer (Phase 3) NCT05566041	(82)
**Metformin**	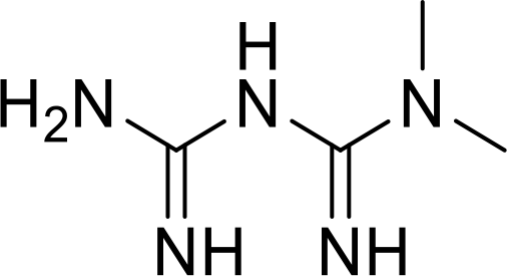	Breast cancer (Preclinical)	(83)
**4-Methylumbelliferone (4Mu)**	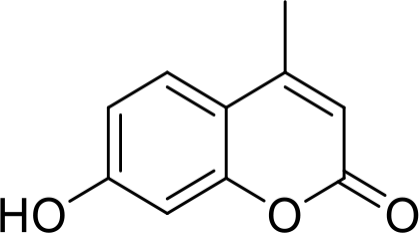	HCC (Preclinical)	(84)
**JQ1**	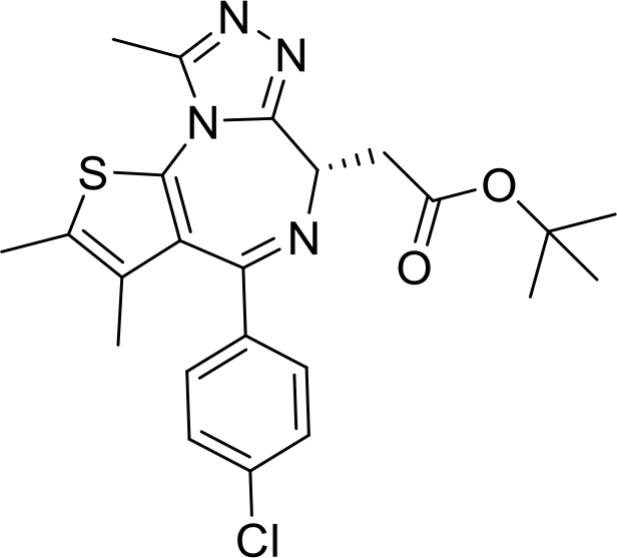	Lymphoma (Preclinical)	(85)
**Gefitinib**	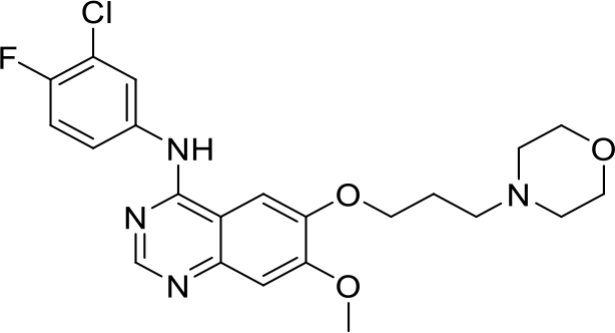	NSCLC (Preclinical)	(86)
**SEN177**	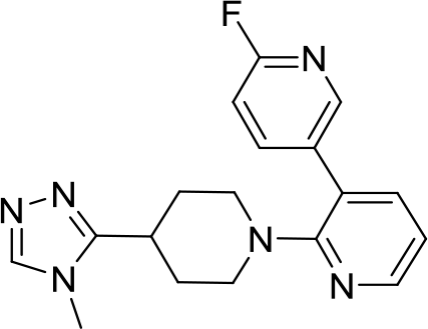	Melanoma (Preclinical)	(87)
**PQ912**	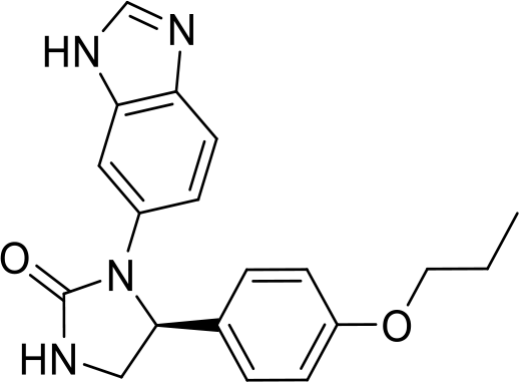	Melanoma (Preclinical)	(87)
**ISM8207**	NA	TNBC (Phase 1)	(88)
NLRP3 Agonist			
**MCC950** **BMS986299**	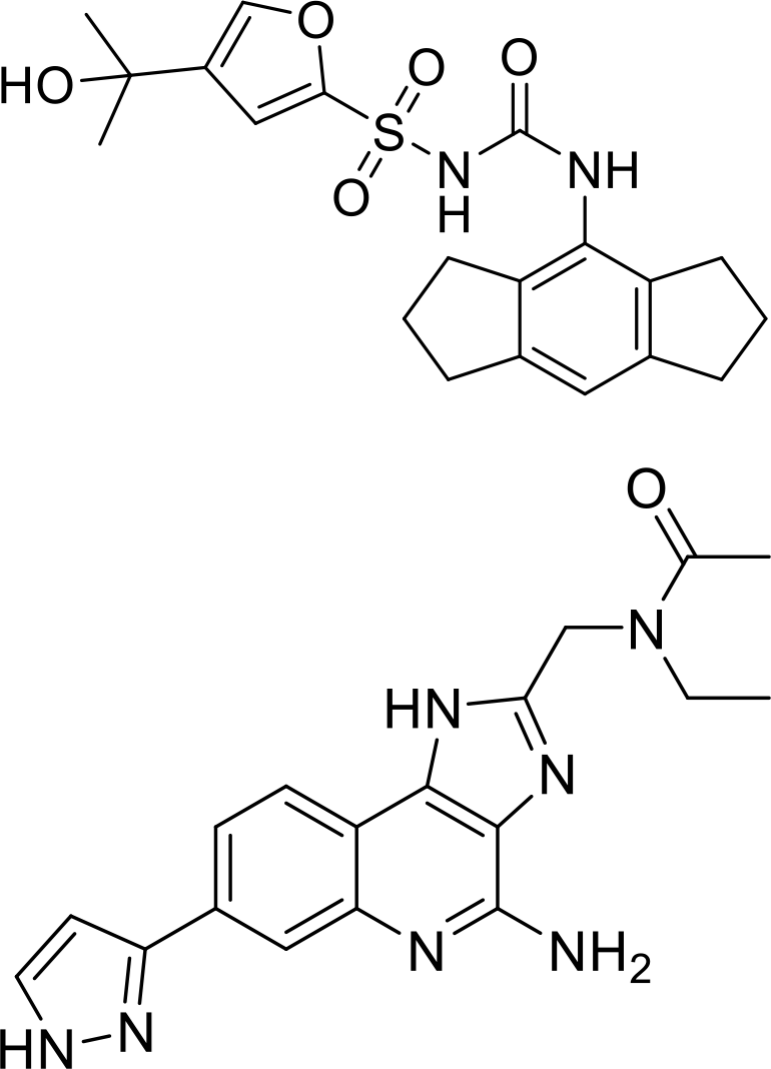	HNSCC (Preclinical)	(89)
		Advanced solid cancers (Phase 1) NCT03444753	
**HEI3090**	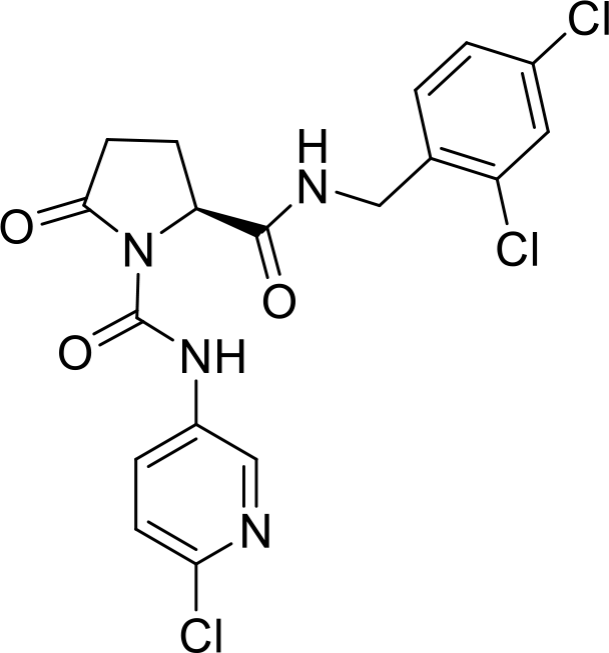	Lewis lung carcinoma (LLC) (Preclinical)	(90)
**Andrographolide (Andro)**	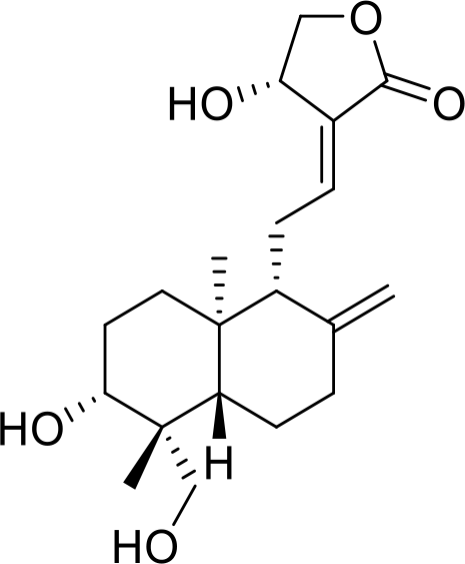	colitis-associated cancer (CAC) (Preclinical)	(91)
TLR agonists			
**Imiquimod** **(TLR7)**	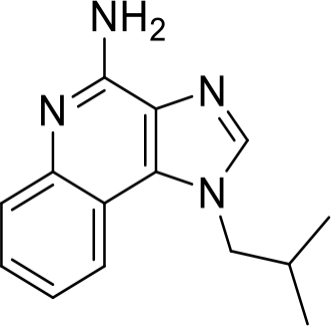	Basal cell carcinoma (Marketed) Lentigo Maligna Melanoma (Head or Neck); Phase III	NCT00314756 NCT01720407
**Guretolimod** **DSP-0509** **(TLR7)**	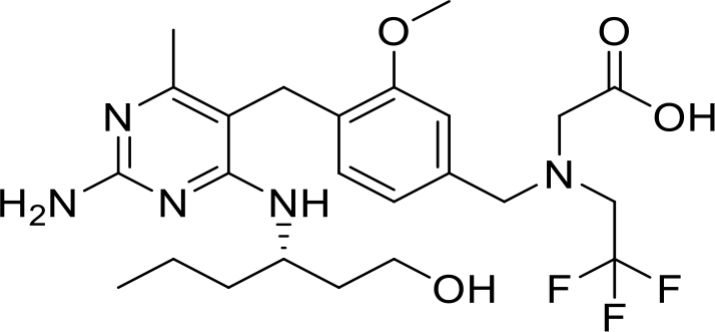	Neoplasms (Phase I/II)	NCT03416335
**JNJ-** **64794964** **(TQ-A3334; TLR7)**	Not disclosed	NSCLC (Phase I/II)	NCT04273815
**LHC-165** **(TLR7)**	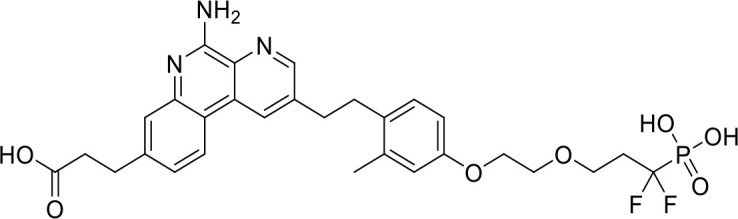	Neoplasms (Phase I/Ib)	NCT03301896
**Telratolimod** **MEDI-9197** **(TLR 7/8)**	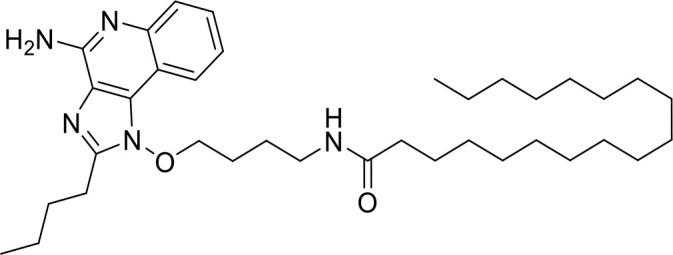	Solid tumors (Phase I)	NCT02556463
**Motolimod** **VTX-2337** **(TLR8)**	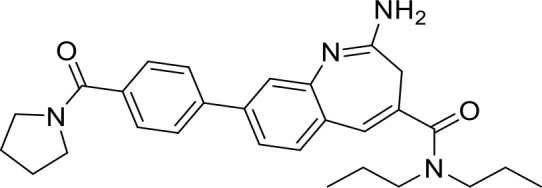	Ovarian cancer (Phase I/II)	NCT02431559
**GSK1795091** **CRX-601** **(TLR4)**	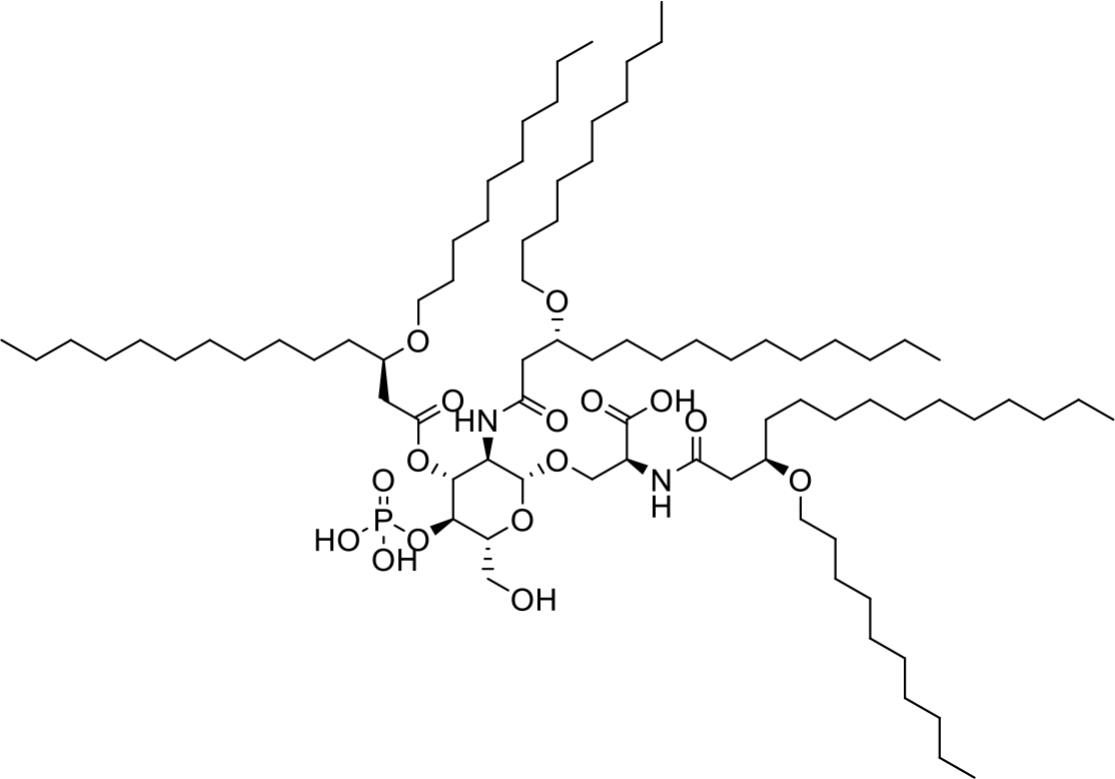	Solid tumors (Phase I)	NCT02798978
**24e** **(TLR 7/8)**	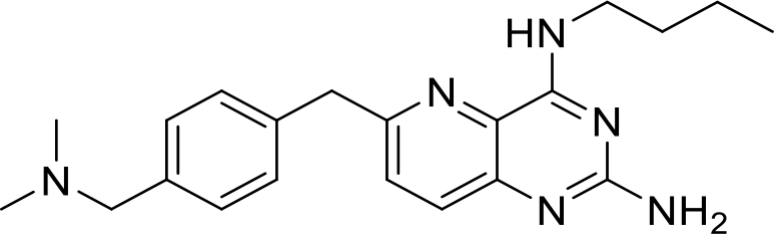	Preclinical	(92)
**CUCPT17e** **(TLR3/8/9)**	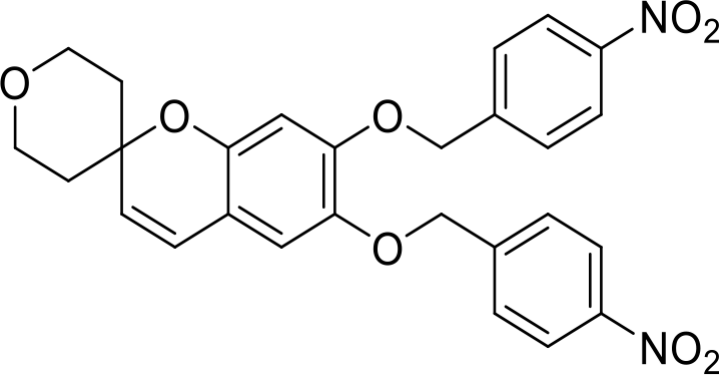	Preclinical	(93)
**SMU-C80** **(TLR1/2)**	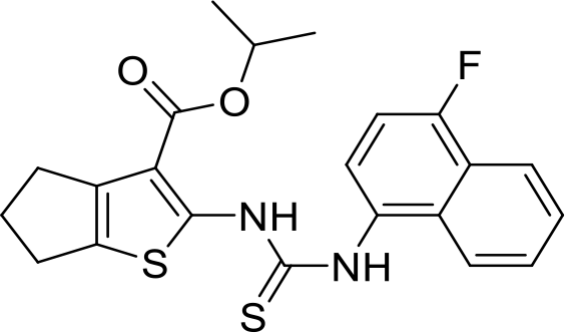	Preclinical	(94)
ENPP1 Inhibitors			
**α-Borano-β,γ-methylene-ATP**	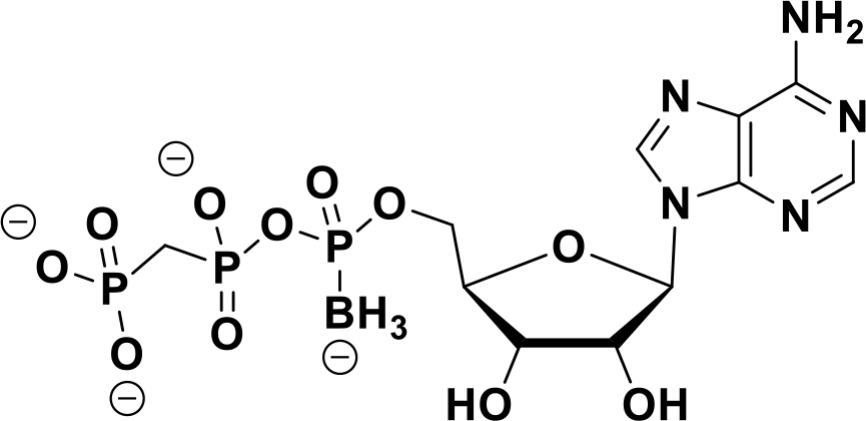	Preclinical	(95)
**α,β-methylene-γ-thio-ATP**	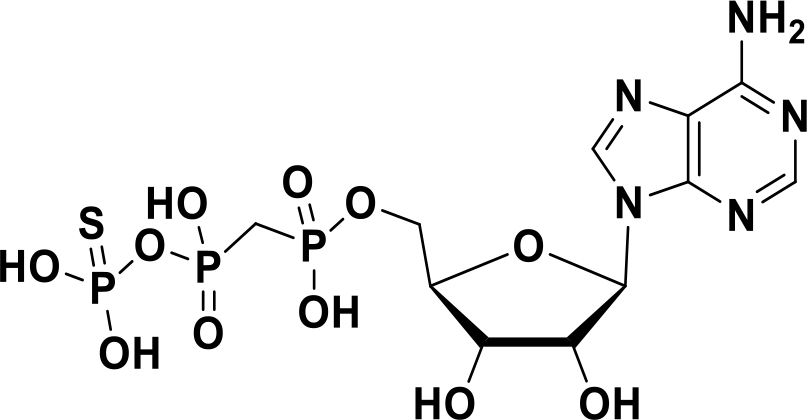	Preclinical	(96)
**4i**	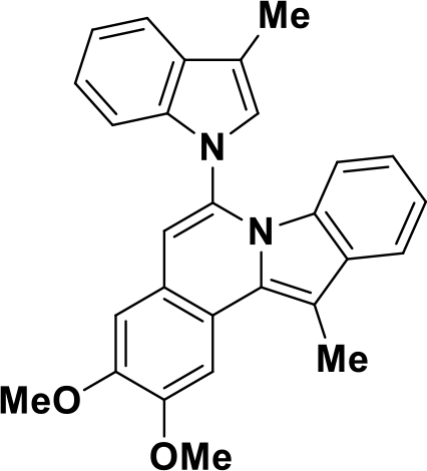	Preclinical	(97)
**5g**	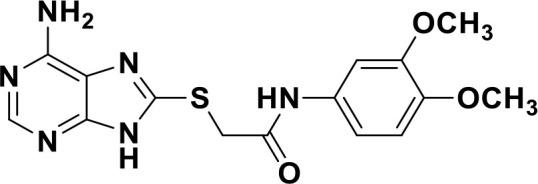	Preclinical	(98)
**QPS1**	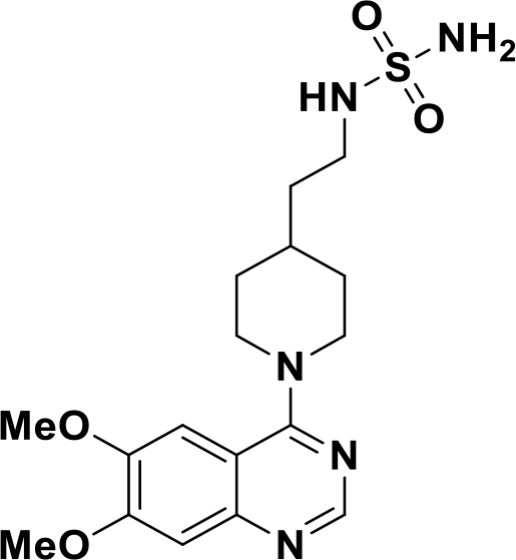	Preclinical	(99)
**7c**	Not disclosed	Preclinical	(100)
**STF-1084**	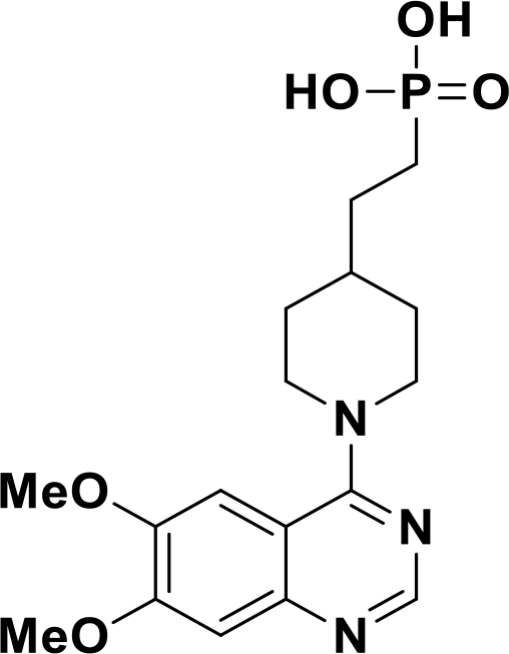	Breast cancers (Preclinical)	(101)
**STF-1623**	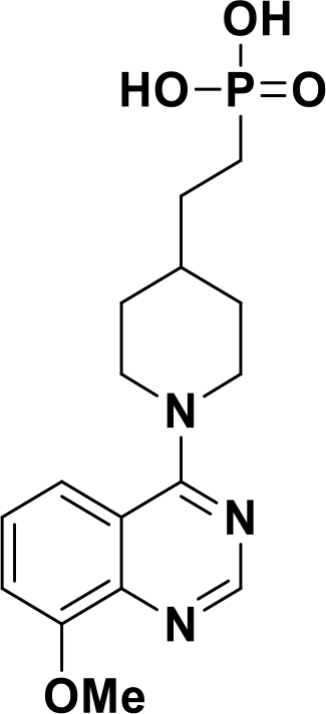	Pancreatic cancers (Preclinical)	(101)
**MV-626**	Not disclosed	Pancreatic cancers, Colon cancer (Preclinical)	(102)
**AVA-NP-695**	Not disclosed	Breast Cancer (Preclinical)	(103)
**SR-8314**	Not disclosed	Melanoma, Colon cancer (Preclinical)	(104)
**ZXP-8202**	Not disclosed	Colon cancer (Preclinical)	(105)
**ZX-8177**	Not disclosed	Colon cancer (Preclinical)	(106)
**ISM5939**	Not disclosed	Colon cancer (Preclinical)	(107)
**RBS2418**	Not disclosed	Advanced unresectable, recurrent or metastatic tumors; (Phase 1, NCT05270213)	(108, 109)
**TXN10128**	Not disclosed	Locally advanced (unresectable) or metastatic solid tumors (Phase 1, NCT05978492)	(110)
**SR-8541A**	Not disclosed	Advanced/​Metastatic Solid Tumors (Phase 1, NCT06063681)	(111)

## Small molecule STING agonists

STING agonists activate the STING pathway, leading to the production of type I interferon and proinflammatory cytokines, resulting in anti-tumor immunity (112, 113). Vadimezan (DMXAA) – A vascular disrupting agent was the first STING agonist extensively evaluated pre-clinically. DMXAA demonstrated a potent anti-tumor immune response in murine tumor models when administered intratumorally (114). However, the lack of translation of this efficacy in clinical trials led to the termination of the trial (115). A detailed study later showed that DMXAA is a direct ligand for murine STING but does not bind to human STING (116). Several cyclic dinucleotides based (CDN) compounds such as ADU-S100, TAK-676, MK1454, BMS-986301, SB11285, BI-1387446, IMSA-101, etc. were the first generation of STING agonist being developed (**Table 1**). These CDN compounds progressed to clinical trials in accessible solid tumors amenable to intratumoral/intravascular/intramuscular delivery. However, their poor ADME properties and stability, lack of substantial anti-tumor effects in the clinical trials and risks of cytokine release syndrome led to discontinuation of several of the trials (117). Recent years have witnessed the development of several small molecule non-CDN based STING agonists (**Table 1**). These possess novel pharmacophores to address the ADME, PK and stability concerns associated with the first-generation CDN based compounds (113). Ramanjulu et al. group reported the amidobenzimidazole (ABZI) as a non CDN based STING agonist with systemic activity in mice (75). ABZI binds in the cGAMP binding pocket with two bound molecules per STING dimer with each ABZI molecule close in space, projected across the STING dimer interface and lacked interactions with the protein. Subsequently, SAR modification at N1-hydroxyphenethyl moiety with a linker between the two molecules to identify a dimeric amidobenzimidazole ligand (diABZI), which showed substantial increase in binding affinity. diABZI induced dose dependent activation of STING and secretion of IFN-β (EC50 = 130 nM). Compound 3 demonstrated satisfactory plasma concentrations and significant antitumor efficacy at a dose of 1.5 mg/kg intravenously in a CT-26 tumor model (75). Pan et al. group identified MSA-2 (benzothiophene- oxobutanoic acid) as an orally bioavailable non-nucleotidic human STING agonist. MSA-2 showed dose dependent anti-tumor effect by intratumoral, subcutaneous or oral administration *in-vivo* which synergized with anti-PD1 therapy (76). Molecular mechanism of action studies revealed that in solution, MSA-2 exists in equilibrium as a non-covalent dimer of MSA-2. Therefore, a series of covalently linked MSA-2 dimers were synthesized and compound 3 has shown enhanced binding affinity to STING (IC50 = 23 ± 7 nM; cGAMP displacement assay) and (EC50: 70 ± 50 nM; Secretion of IFN- β) (76). Chin et al. group identified SR-717 as a non-nucleotide STING agonist (EC50 = 2.1 µM) that demonstrated potent antitumor activity and promoted the activation of CD8+ T, NK and dendritic cells *in vivo (* 77). Co-crystal structure revealed that SR-717 induced cGAMP mimetic closed conformation with STING. Moreover, SR-717 also induced the expression of clinically relevant targets, including PD-L1 in a STING-dependent manner (77). Banerjee et al. recently discovered CRD-5500 (Bicyclic benzamides; Structure not available) as a non-nucleotide STING agonist that activates major human STING variants and generates a strong pro-inflammatory response (78). CRD-5500 demonstrated antitumor activity *in-vivo* and a synergistic effect with anti-PD1 (78). Several other groups have reported Non-CDN based small molecule STING agonists (**Table 1**)). However, systemic administration of STING agonists causes cytokine release syndrome (immunotoxicity) due to the ubiquitous expression of STING in normal and tumor tissues which impedes its clinical translation. In order to minimize the risk of toxicity, tumor targeted delivery of STING agonists using Antibody- drug conjugates (ADC) are being explored as potential cancer immunotherapeutics. Chen et al. group reported STING ADC (αEGFR-172ADC) for their molecule IMSA172 that demonstrated potent antitumor efficacy *in-vivo* and promoted activation of dendritic cells, T cells, natural killer cells, natural killer T cells, as well as promotion of M2 to M1 polarization of tumor-associated macrophages. The STING ADC also showed synergistic effect with anti-PD-L1 (80). ADCs have emerged as a promising alternative to address cytokine related toxicity concerns, and extensive work is currently ongoing which is beyond the scope of this review.

## ENPP1 inhibitors

ENPP1 has emerged as a promising therapeutic target in cancer immunotherapy and inhibition of ENPP1 augments anticancer immunity via STING-mediated innate immune activation (46, 101, 118–120). Nucleotide-based ENPP1 inhibitors have been developed (**Table 1**); however, these possess low oral bioavailability, high acidity of the phosphate backbone, moderate stability and potential off-target biological effects (119). To address the existing concerns several groups have identified and developed series of non-nucleotide based ENPP1 inhibitors (**Table 1**). An isoquinoline derivative based ENPP1 inhibitor has been reported by Ausekle et al. with IC_50_ values of 0.36 μM using p-NP-TMP as a substrate (97). Chang et al. reported a thioacetamide inhibitor with K_i_ value of 5 nM against human ENPP1 using p-Nph-5’-TMP as substrate (98). Patel et al. reported a quinazoline-piperidine-sulfamide ENPP1 inhibitor (QS1) with an IC_50_ of 36 nM using ATP as a substrate (121). Carozza et al. designed a phosphonate analog of QS1, STF-1084 with Ki,_app_ = 110 nM. A quinazoline-4-piperidine sulfamide compound, QPS1 with Ki = 59 ± 5 nM is reported by Patel and coworkers as a selective non-competitive ENPP1 inhibitor (121). Using QPS1 as the lead compound, Forcellini et al. developed quinazoline-4-piperidine sulfamide analogues based ENPP1 inhibitors with K_i_ < 105 nM against human ENPP1 (100). STF-1084 is a potent, specific, and cell impermeable ENPP1 inhibitor (42). Another cell-impermeable, nontoxic specific ENPP1 inhibitor is STF-1623 (Ki,_app_ = 16 nM in an *in vitro* assay) which delayed tumor growth in Panc02 syngeneic, pancreatic tumor model. STF-1623 resulted in a decreased rate of locoregional failure of breast cancer models treated with surgery and radiation (122).

Other small molecule ENPP1 inhibitors with promising preclinical results have been reported (**Table 1**), however, the structure for these inhibitors have not been disclosed yet. MV-626 is a selective ENPP1 inhibitor developed by Mavupharma (Ki: 5 nM) with 100% oral bioavailability in rats and mice. *In-vivo*, MV-626 in combination with radiation treatment delayed tumor growth in syngeneic Panc02 tumor model. Further, the combination of MV-626 with anti-PD-L1 enhanced overall survival in the MC38 murine colon carcinoma model (102). Another preclinical candidate AVA-NP-695, from Avammune Therapeutics is a selective and highly potent ENPP1 inhibitor, which apart from its immunomodulatory effect also modulates cancer metastasis. AVA-NP-695 has demonstrated strong synergy with PARPi, resulting in superior tumor growth inhibition and reduced metastasis in syngeneic 4T1 breast cancer mouse models (103, 123). SR-8314 is a potent ENPP1 inhibitor with a K_i_ value of 0.079 µM, which increases IFNβ, ISG15 and CXCL10 expression in THP1 cells*. In-vivo* efficacy of SR-8314 was confirmed in syngeneic murine tumor model and increased levels of CD3, CD4, and CD8 T cells were observed in SR-8314 treated tumors (104). ENPP1 inhibitor, ZXP-8202 with EC_50_ value of 20 nM in cell based-assay and ZX-8177 with IC_50_ of 9.5 nM are reported with 37-60% tumor growth inhibition (TGI) for ZX-8177 in CT26 syngeneic mouse model. Synergistic tumor inhibition of ZX-8177 in combination with anti-PD-L1 in MC38 syngeneic mouse model (TGI of anti-PD-L1 antibody vs. combo treatment is 53% vs. 75%) was observed as well. (106). ISM5939 is a selective inhibitor of ENPP1 with IC_50_ of 0.63 nM in the enzymatic assay with ISM5939 monotherapy (30 mg/kg, p.o. BID) having a 67% TGI in MC38 model (107).

A few ENPP1 small molecule inhibitors have advanced to early clinical stages for potential use as cancer immunotherapeutics that enhance the STING-mediated immune response (**Table 1**). The structures of most of these compounds have not been disclosed yet. In 2022, the first Phase I clinical trial was initiated for an orally available small molecule ENPP1 inhibitor, RBS2418 from Riboscience in combination with pembrolizumab or as a monotherapy for advanced unresectable, recurrent or metastatic tumors (108, 109). TXN10128 is another orally available ENPP1 inhibitor in Phase 1 currently being evaluated for solid tumors (NCT05978492). The IC_50_ value of selective ENPP1 inhibitor TXN10128 with cGAMP as substrate was 4 nM. TXN10128 in combination with anti-PD-L1 antibody inhibited tumor growth in MC38 syngeneic mouse model (110). SR-8541A is an oral ENPP1 inhibitor candidate from Stingray Therapeutics in Phase 1, being evaluated in subjects with advanced/metastatic solid tumors (NCT06063681). *In vitro* SR-8541A triggers strong immune responses, with increased expression of IFN-β, ISG15 and CXCL10 and showed decreased tumor growth in a CT26 colon cancer model (124).

## Small molecule TLR agonists

Toll-like receptors (TLRs) are pattern recognition receptors (PRRs) involved in the regulation of innate immunity. TLRs play a pivotal role in recognizing pathogen-associated molecular patterns (PAMPs) or damage-associated molecular patterns (DAMPs) and initiating immune responses. The activation of specific TLRs like TLR1/2, TLR3, TLR5, TLR7/8 and TLR9 have previously demonstrated antitumor immune responses. Moreover, TLR agonists like Diprovocim (TLR1/2) (125), 1V270 (TLR7) (22) and SD-101 (TLR9) (126) have shown synergistic effects with immune checkpoint inhibitors thus enhancing the tumor immunogenicity (**Table 1**). Therefore, TLR agonists present a promising strategy for cancer immunotherapy (127, 128). Imiquimod is a small molecule TLR7 agonist being studied in clinical trials for various tumors such as basal cell carcinoma (NCT00314756) and melanoma (NCT01720407) (129). Several other TLR7 agonists are at different stages of clinical development including Guretolimod (DSP-0509; NCT03416335; Phase I/II) for the treatment of solid tumors administered as monotherapy and in combination with pembrolizumab (130). Others include JNJ-64794964 (TQ-A3334; NCT04273815; Phase IB) for the treatment of advanced non-small cell lung cancer (131) and LHC-165 (NCT03301896; Phase I/IB; Terminated) as monotherapy and in combination with PDR001 in patients with advanced malignancies (132). Telratolimod (MEDI-9197; NCT02556463) is a TLR7/8 agonist being studied in solid tumors as monotherapy or in combination with durvalumab and/or palliative radiation therapy but failed (**Table 1**) (133). Motolimod (VTX-2337; NCT02431559; Phase I/II) is a TLR8 agonist being evaluated in combination with durvalumab and pegylated liposomal doxorubicin for recurrent, platinum-resistant ovarian cancer (**Table 1**) (134). GSK1795091 (CRX-601; NCT02798978; Phase I) is a TLR4 agonist being evaluated for safety, tolerability, pharmacodynamic (PD), and pharmacokinetics (PK) profile determination in healthy subjects (135). Wang et al. reported compound 24e (pyrido[3,2-d]pyrimidine-based) as TLR 7/8 dual agonists with EC50 of 24 nM hTLR 7 and 10 nM hTLR 8 (92). Compound 24e induced the secretion of IFN-α, IFN-γ, TNF-α, IL-1β, IL-12p40, and IP-10 in human peripheral blood mononuclear cell assays. Compound 24e also showed significant tumor growth inhibition as monotherapy in the CT-26 mouse model which led to complete tumor regression when combined with anti-PD-L1 antibody (92). Zhang et al. reported compound 17 e (CU-CPT17e) as multi-TLR agonist that shown to activate TLR 3, 8 and 9. It induces a strong immune response via the release of various cytokines in THP-1 cells. It has also inhibited the growth of HeLa cancer cells in their *in vitro* assay (93). Chen et al., reported SMU-C80 as TLR1/2 dual agonist with an EC50 of 31 nM. SMU-C80 is reported to act through the MyD88 and NF-κB pathways and mediated cytokine release and the activation of immune cells to produce antitumor effect *in vitro (* 94).

## CD47-SIRPα interaction blocker

Overexpression of CD47 enables tumor cells to evade immune surveillance via the blockade of phagocytic mechanisms and is associated with poor survival in various cancer (136, 137). Therefore, inhibition of CD47-SIRPα axis has a significant impact on tumor immunotherapy (138). NCGC00138783 is a small-molecule inhibitor that directly blocks the binding of CD47 to SIRPα with an IC50 of ~50 μM (**Table 1**) (81, 139). Further optimization of its physicochemical properties as well as its druggability led to the discovery of its analogues, NCGC00138419 and NCGC00138430 (139). Small-molecule inhibitors that inhibit CD47 expression at the transcriptional and translational levels are RRx-001, 4-methylumbelliferone (4-Mu), JQ1, and gefitinib. RRx-001 suppressed CD47 expression in tumor cells through the inhibition of c-MYC, a positive regulator of CD47 (140, 141). 4-Methylumbelliferone (4Mu) is a hyaluronan synthesis inhibitor that downregulated the expression of CD47 and promoted phagocytosis of intraperitoneal macrophages of hepatocellular carcinoma (HCC) cells (84). Combination of 4Mu with adenovirus encoding IL-12 (AdIL-12) significantly reduced tumor volume (TGI ∼80%) and largely improved overall survival in an orthotopic HCC mice model (84). JQ1 is a small-molecule inhibitor of bromodomain and extra-terminal (BET) proteins. JQ1 suppresses expression of CD47 and PD-L1 in acute lymphoblastic leukemia, melanoma, and non-small cell lung cancer (NSCLC) cells through a c-MYC-mediated pathway (85, 142). An interesting report had highlighted that the role of Glutaminyl cyclase isoenzyme (QPCTL) is critical for pyroglutamate formation on CD47 at the SIRPα binding site (87) and inhibition of QPCTL activity enhances antibody-dependent cellular phagocytosis and cellular cytotoxicity of tumor cells. QPCTL inhibitors, SEN177 (IC50 = 0.013 μM for QPCTL) and PQ912 have been reported for their impact on the CD47-SIRPα interaction (87, 143). In silico Medicine in collaboration with Fosun Pharma developed a first-in-class orally available small molecule ISM8207 (IC50 < 0.5 nM in enzymatic assay) inhibitor of QPCTL. ISM8207 has advanced to Phase I clinical trials for the treatment of triple-negative breast cancer (TNBC) and B-cell non-Hodgkin lymphoma (88).

## NLRP3 modulators

Large cytosolic multiprotein complexes known as inflammasomes are involved in the host defence against microbial infections by mediating important inflammatory innate immune responses. NLRP3, the best-studied NLRP, is expressed by DCs, lymphocytes, macrophages, and non-immune populations such as epithelial cells. In various preclinical and clinical studies, the role of NLRP3 has been extensively explored and a correlation between lower expression of NLRP3 and advanced cancer has been established. Interestingly, both NLRP3 agonists and inhibitors have shown anti-tumor efficacy (**Table 1**). The novel NLRP3 agonist BMS-986299 is currently undergoing evaluation in a phase I clinical trial, both as a standalone treatment and in combination with nivolumab and ipilimumab, for advanced solid tumors [NCT03444753] (144). In a murine model of HNSCC, a novel NLRP3 inhibitor known as MCC950 demonstrated the ability to delay tumor growth, decrease levels of MDSCs, Tregs, and TAMs, while also enhancing T cell infiltration within the tumor microenvironment (TME). Additionally, Douguet et al. demonstrated an alternate way to activate the NLRP3 inflammasome is via P2RX_7_’s activation (145). HEI3090, a pyrrolidin-2-one derivative is a positive modulator of the P2RX_7_ receptor that potentiates anti-PD-1 treatment to effectively control the growth of lung tumors. This demonstrated complete tumor regression in 80% of LLC tumor-bearing mice, works by activating the NLRP3 pathway (90). A small molecule andrographolide (Andro) protected mice against colitis-associated cancer (CAC) in the mouse model of (AOM)-dextran sulphate sodium (DSS) through inhibiting NLRP3 inflammasome activation in macrophages. The tumor size was reduced by Andro in a dose-dependent manner and the average tumor load was significantly decreased in the Andro-treated group (91).

## Discussion

Developing small molecule immunomodulators for the innate immune system for the treatment of cancer is a fast growing and active area of research and development. The innate immune system plays a crucial role in recognizing and eliminating cancer cells. While small molecule immunomodulators offer several advantages, they also come with certain disadvantages and challenges. Small molecules may interact with unintended targets, leading to off-target effects. This lack of specificity can contribute to adverse reactions and limit the therapeutic index. For e.g., one challenge in the development of STING agonists is the potential for systemic toxicity. Activating the STING pathway can induce a robust immune response, and balancing this response to avoid excessive inflammation and off-target effects is critical. STING pathway hold significant potential in combination modalities like radiation and chemotherapies which induces double stranded DNA breaks. Alternatively, ENPP1 inhibition has much more potential to exert a significant anti-tumor activity with reduced autoimmune related toxicity concerns. Recent reports have also suggested that the loss of ENPP1 function in both cancer cells and normal tissues resulted in a reduced primary tumor initiation and growth. The phenomenon also hindered metastasis through a mechanism that relies on extracellular cGAMP and is dependent on STING (46). Notably, breast cancer patients exhibiting reduced ENPP1 expression demonstrate elevated levels of immune infiltration. These patients show enhanced responsiveness to therapeutics affecting cancer immunity at points preceding or following the cGAMP-STING pathway, such as PARP inhibitors and anti-PD1 treatments. Currently, three phase 1 clinical trials are ongoing with ENPP1 inhibitors and the potential of these inhibitors will be analyzed once the study completes. Apart from the STING pathway, other innate immune pathways also hold significant potential in cancer immunotherapy. TLR agonists are used as adjuvants to enhance the effectiveness of cancer vaccines or as standalone agents to stimulate the innate immune response against tumors.

Since, innate and adaptive immunity is highly interconnected, innate immune modulators can play a crucial role in overcoming immune tolerance and avoid resistance being developed against immune checkpoint inhibitors. Extensive research is currently ongoing to evaluate the combination of small-molecule immunotherapies with various anticancer modalities, as well as the monotherapy of these agents. For e.g. resistance to Immune checkpoint inhibitors (ICIs) therapy is frequent, and approximately only 30% to 40% of patients benefit from ICIs. ICIs, particularly monoclonal antibodies that specifically target CTLA-4 and PD-1/PD-L1, have greatly improved patient outcomes and progressed cancer treatment. Evidence suggests that combination of small molecule innate immune modulators with ICIs and other therapeutic approaches may greatly increase therapeutic efficacy. With the growing evidence of several immune checkpoint proteins, efforts need to be made towards development of small molecule inhibitors against these immune checkpoints. With growing research and early phase clinical trial along with biomarker driven study designs, novel pathways and combination strategies will be identified. Future developments in cancer immunotherapy appear to be greatly promising when it comes to exploiting these synergistic combination pathways that simultaneously engage innate and adaptive immunity.
